# Access to Scientific Publications: The Scientist's Perspective

**DOI:** 10.1371/journal.pone.0027868

**Published:** 2011-11-17

**Authors:** Yegor Voronin, Askar Myrzahmetov, Alan Bernstein

**Affiliations:** Global HIV Vaccine Enterprise, New York, New York, United States of America; National Institute of Health, United States of America

## Abstract

**Background:**

Scientific publishing is undergoing significant changes due to the growth of online publications, increases in the number of open access journals, and policies of funders and universities requiring authors to ensure that their publications become publicly accessible. Most studies of the impact of these changes have focused on the growth of articles available through open access or the number of open-access journals. Here, we investigated access to publications at a number of institutes and universities around the world, focusing on publications in HIV vaccine research – an area of biomedical research with special importance to the developing world.

**Methods and Findings:**

We selected research papers in HIV vaccine research field, creating: 1) a first set of 50 most recently published papers with keywords “HIV vaccine” and 2) a second set of 200 articles randomly selected from those cited in the first set. Access to the majority (80%) of the recently published articles required subscription, while cited literature was much more accessible (67% freely available online). Subscriptions at a number of institutions around the world were assessed for providing access to subscription-only articles from the two sets. The access levels varied widely, ranging among institutions from 20% to 90%. Through the WHO-supported HINARI program, institutes in low-income countries had access comparable to that of institutes in the North. Finally, we examined the response rates for reprint requests sent to corresponding authors, a method commonly used before internet access became widespread. Contacting corresponding authors with requests for electronic copies of articles by email resulted in a 55-60% success rate, although in some cases it took up to 1.5 months to get a response.

**Conclusions:**

While research articles are increasingly available on the internet in open access format, institutional subscriptions continue to play an important role. However, subscriptions do not provide access to the full range of HIV vaccine research literature. Access to papers through subscriptions is complemented by a variety of other means, including emailing corresponding authors, joint affiliations, use of someone else's login information and posting requests on message boards. This complex picture makes it difficult to assess the real ability of scientists to access literature, but the observed differences in access levels between institutions suggest an unlevel playing field, in which some researchers have to spend more efforts than others to obtain the same information.

## Introduction

Sharing the results of research through peer-reviewed publication is intrinsic to the scientific process and to progress in science. It is also widely appreciated that access to scientific findings can lead to important advances in health care by providing the evidence upon which to base sound public policy, and by bringing together researchers from the developed and developing world, thereby fostering new collaborations and building capacity. Moreover, it is in everyone's interests if the public, which provides a large proportion of the funds required for scientific research through taxes, and many of whom chose to volunteer for clinical trials, is actively aware of and engaged in the activities of the research community. Although the public can't be expected to have the same technical expertise as the scientific community, an engaged public that knows that the scientific community is accountable and transparent will likely be more supportive of research and will be more likely to agree to serve as trial volunteers [1]. For these reasons, various global health organizations and alliances have called for both increased access and sharing of research data and primary publications [2]–[4]. The 2010 Scientific Strategic Plan of the Global HIV Vaccine Enterprise states that the field should “seek consensus on the principle of rapid access to data and develop a global approach to develop the infrastructure required to annotate, deposit, and analyze the increasingly large and complex amounts of laboratory, clinical and population data generated in the search for an HIV vaccine”[4].

Recent advances in information technology and computational techniques have created new opportunities to share both data and publications rapidly. At the same time, these *in silico* advances have been matched by newer technologies capable of generating very large and complex datasets (e.g. next generation sequencing, ‘omics technologies and other high-throughput approaches). There is, therefore, both an urgent need and an opportunity for a harmonized infrastructure and culture for the sharing of data and publications. The volume of scientific publications has been growing at an exponential rate and is stressing the budgets of university libraries, a situation referred to as the ‘serials crisis’ [5]. At the same time, the globalization of science and increasing involvement of researchers from the developing world in the scientific enterprise makes it imperative that scientific information be available on a level playing field throughout the world. The revolution in information technology and specifically the development of the web has revolutionized scientific publishing itself, making it possible to disseminate papers anywhere in the world, easily, inexpensively and rapidly.

Rapid access to data and publications is of particular importance to the developing world. Infectious diseases such as HIV, malaria, and tuberculosis disproportionately affect people in the developing world - out of 33 million people estimated to be living with HIV in the world, 26 million live in sub-Saharan Africa and South-East Asia [6]. Over $800 million are invested annually in HIV vaccine research, making it one of if not the largest single area of research investment for diseases affecting the developing world [7]. Yet the overwhelming majority of research funds and researchers come from North America and Europe. Because the outcomes of HIV vaccine research are of critical importance for Africa and Asia, not only for scientists in those regions, but also for health professionals, regulators, policy makers and affected communities, it is important to evaluate how research findings are disseminated. The area of HIV vaccine research, similar to other biomedical fields, saw recent growth in the popularity of open-access journals, such as *PLoS Pathogens* and *Retrovirology*, but the exact impact of these changes is not yet clear. For these reasons, we set out to use the field of HIV vaccine research as a barometer to measure the degree to which scientists have access to published research that is of critical importance in this fast-moving field.

Our approach was as follows: We assessed the literature related to HIV vaccine research through two sets of papers. The first set, “recent papers”, was created by searching for “HIV vaccine” in the NCBI database on December 6, 2010 and selecting the 50 most recently published articles, checking for relevance and excluding special-issue publications containing collections of articles. The second set, “cited papers”, was created by randomly selecting 20 papers from the first set and then randomly selecting 10 citations from each of those papers, resulting in a dataset of 200 papers. We assessed the ability of researchers at a number of institutions around the world to access both sets of papers online either by reviewing lists of institutional subscriptions (when available) or by directly asking researchers to attempt to download these papers.

## Results

### Open Access

**Figure 1:**
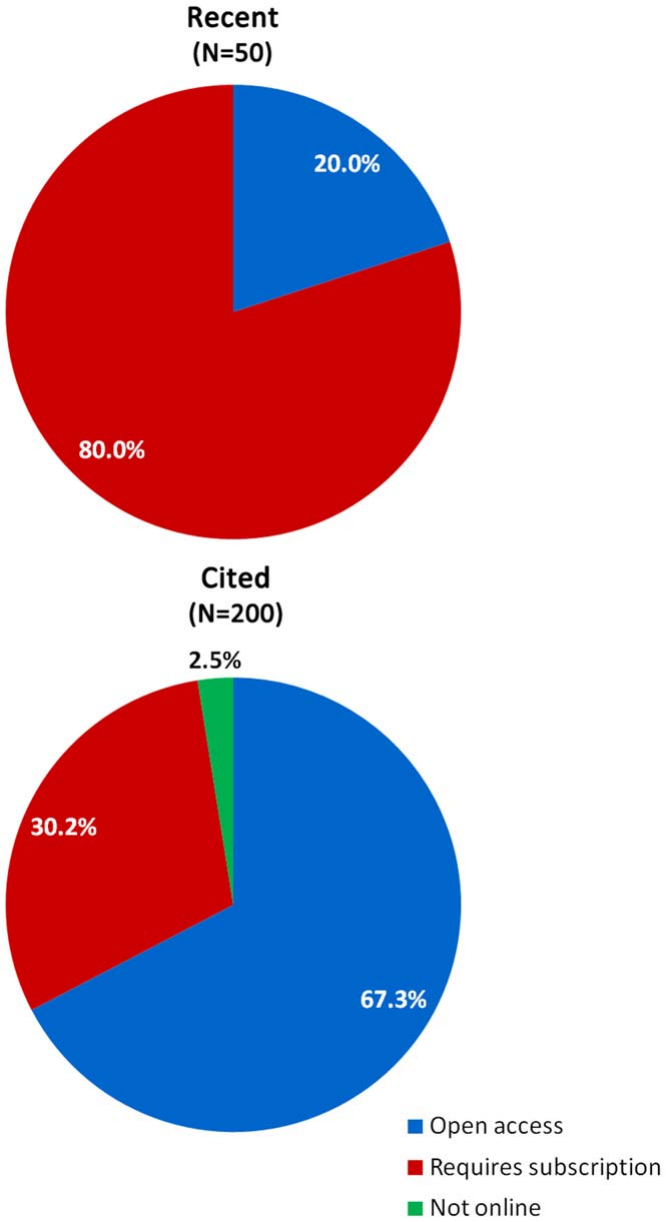
Availability of HIV vaccine research articles online. Two randomly selected sets of papers were created and checked for availability online. Most of the recently published papers required subscription for access, while the “cited” set of papers (randomly selected from citations within the “recent” set) were more accessible, presumably due to journals moving articles from subscription-restricted content into open access. A small proportion of cited articles were not available online.

In the recent Study of Open Access Publishing (SOAP) conducted by the European Commission, 89% of scientists viewed open access as beneficial and conducive to scientific progress, but at the same time only 25% were willing to publish in open access journals [8]. Reviewing our “recent” set of papers, we found that only 20% were available in open access upon publication (Fig. 1).

Grant policies of many funding organizations, including the NIH (the largest funder of HIV vaccine research), require that papers resulting from their financial support become freely available within 12 months after publication [9]. However, many subscription-based scientific journals go beyond this requirement by making papers available sooner (6–9 months after publication). In addition, some universities require that research publications by faculty be publicly accessible within a certain period of time. Perhaps as a result of these policies, papers from the “cited” dataset were significantly more accessible than “recent” papers, with 67% being available without a subscription (Fig. 1). Nevertheless, almost one third of publications were not accessible to investigators who do not have a subscription (five papers, or 2.5%, were too old to be available online). From 134 “cited” papers available without a subscription, 117 were available on the journal's website and 12 were available through PubMed Central. Only a small fraction of the publications (5 out of 200 or 2.5%) were deposited on the websites of the authors of these publications or in institutional databases (so called “green road” to access).

### Subscription-based access

Institutes and universities have to make choices when selecting journal subscriptions. These decisions are presumably based on the budget of the library and the research interests of the scientists. As a result, subscription patterns vary widely amongst institutions and do not necessarily provide access to all subscription-based literature. We decided to compare the level of access at several research institutes and universities around the world, focusing on those that are involved in HIV research.

#### United States

**Figure 2:**
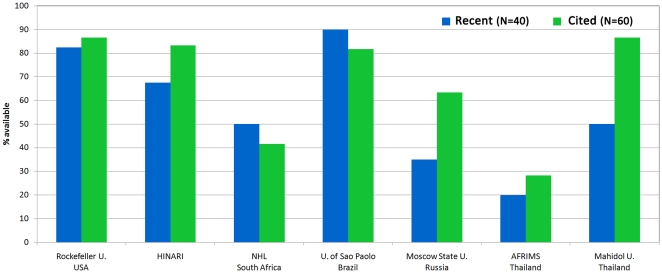
Subscription-based availability of HIV vaccine research articles at a number of institutes and universities around the world. The “recent” and “cited” are the two sets of papers described in the legend to Fig. 1. Only papers which were not available through open access sources, but were available online, were included in this analysis.

We first looked at literature access at Rockefeller University. Rockefeller University has a very active research program, including several laboratories working on HIV, and maintains an extensive library. Interestingly, despite an annual subscription budget of approximately one million dollars, the university still does not have access to all the articles in our datasets. In both “recent” and “cited” sets, only 80–85% of papers requiring subscription were accessible through the Rockefeller University library website (Fig. 2). This observation is a vivid illustration of the “serials crisis” affecting even well funded academic libraries [5].

#### Sub-Saharan Africa

Sub-Saharan Africa is the region most affected by HIV. This region of the world also includes countries with some of the lowest Gross Domestic Product (GDP) per capita. Thus, institutions in this region would not be expected to afford journal subscriptions on par with Rockefeller University or other institutions in North America and Europe. Fortunately, the WHO-supported Health Internetwork Access to Research Initiative (HINARI) provides free access to registered institutions in many countries in the region, including Democratic Republic of Congo, Gambia, Kenya, Malawi, Nigeria, Tanzania, Uganda, Zimbabwe and others [10]. For our sets of papers, the level of access through HINARI matched that of Rockefeller University for “cited” literature (82%) and was only a little lower for “recent” literature (65%) (Fig. 2).

#### Republic of South Africa

We chose to look at the access issue in South Africa for several reasons: First, South Africa has the unfortunate distinction of having one of the highest rates of incidence of HIV in the world. Second, South Africa has an especially strong scientific community by both African and international standards and South African scientists at public institutes and universities actively collaborate with investigators in the North and are involved in a wide array of HIV-related research projects. Third, South Africa is one of the more prosperous countries in the region and hence does not qualify for access through HINARI. As shown in Fig. 2, only 50% of “recent” literature was available through subscriptions at the National Health Laboratory (NHL) and the access to “cited” literature was even lower (42%). Thus, South African institutions, such as the NHL, actually have a lower level of access to the literature than institutions in less-prosperous neighboring countries.

#### Brazil and Russia

The economies of Brazil and Russia (in both absolute numbers and per capita) are similar to each other, and both countries are facing a growing HIV epidemic. Brazil's University of Sao Paolo had the highest level of access among all institutions we surveyed, surpassing Rockefeller University with access to 90% of “recent” papers and to 82% of “cited” papers (Fig. 2). In contrast, Moscow State University, the most prestigious and best-funded university in Russia, had a mediocre level of access to “cited” literature (63%), and access to “recent” literature (35%) was one of the lowest that we observed (Fig. 2). Thus, there was a significant difference in the level of access to the HIV vaccine literature in these two countries.

#### Thailand

We considered two institutions in Thailand, a country which has been actively engaged in HIV vaccine research. Through the Thai Ministry of Public Health, over 16,000 Thai citizens participated as volunteers in RV144, a clinical trial of an HIV vaccine regimen which was the first trial to show partial, transient efficacy in the prevention of HIV transmission [11]. We observed that Mahidol University, the largest university in Thailand, had excellent access to the “cited” literature (86%), but only 50% of “recent” papers were available. In contrast, AFRIMS, a Thai-US collaborative institute of medical research had the lowest level of access to both “recent” (20%) and “cited” (28%) papers (Fig. 2) among institutions that we surveyed. Many researchers at AFRIMS are affiliated with the US-based Walter Reed Institute and, perhaps, are able to access papers through subscriptions at that institute (although we did not evaluate that possibility in this study).

### Requesting reprints

**Figure 3:**
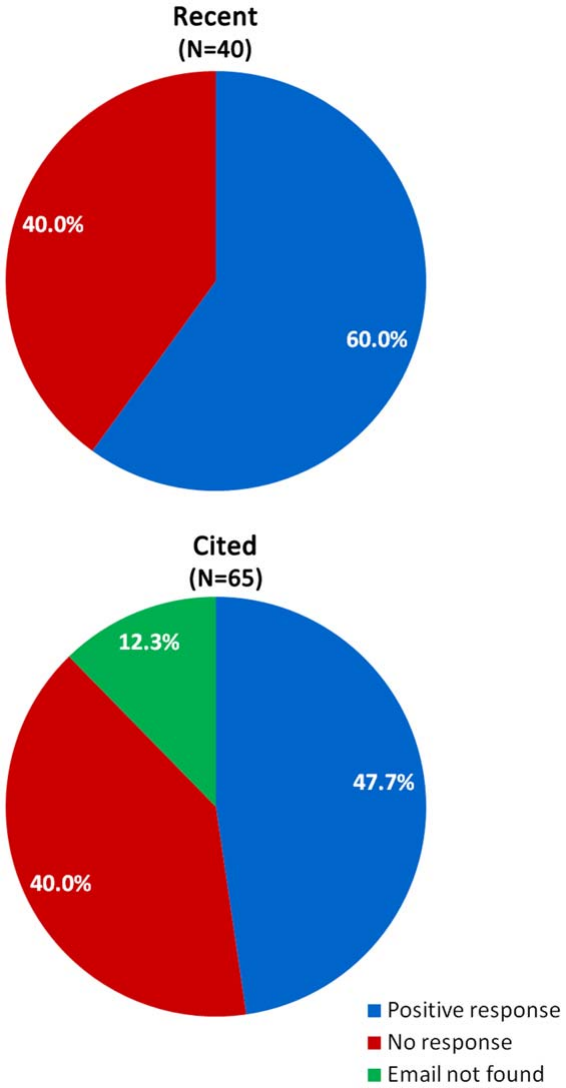
Outcomes of contacting authors with an email request for a copy of the paper. The “recent” and “cited” are the two sets of papers described in the legend to Fig. 1. Emails were obtained from journal's website, NCBI, by web searches and in institution's directories. “Positive responses” include responses with a pdf or Word document of the requested paper attached to the email. “No response” includes bounced emails (due to email address no longer being active), lack of reply and, in one case, refusal to send a copy due to publisher holding the copyright. “Email not found” includes articles for which the email of the corresponding author could not be located.

In the past, before the growth of the internet, requesting a reprint from authors via post mail was a common way to obtain a copy of a paper. We investigated whether such requests, but now through email, can compensate for the lack of institutional subscription. We contacted the corresponding author (if information on corresponding author was not available, we assumed that it was the last author) with a request for a reprint using a sender email address not affiliated with any institution and with a name unknown to the authors in order to mirror a common situation in which the researcher is not familiar with the person requesting the article. For “recent” papers, we sent out 40 requests and received 24 positive responses (60% success rate) (Fig. 3). For “cited” papers, we intended to send out 65 requests, but could not locate the email addresses of eight of the corresponding authors. For the remaining 57, requests were sent out and 31 authors responded by sending a copy of the paper (54% success rate). Among the 26 emails that were unsuccessful, 8 email addresses were outdated, one author declined to provide the paper citing copyright issues with the journal and the rest did not respond. The two thirds of those who replied to the request did so on the same day or the next. However, the other third of respondents took on average 11 days to reply (median 3 days, maximum 54 days).

## Discussion

In this study, we have presented a data-driven, global comparison of access to the scientific literature. We chose to focus our attention on HIV vaccine research - an area of intense research activity with special relevance for the developing world. We found that 20% of recent articles in this field were published in open-access journals. This proportion is a larger fraction than the 10% found in the recent SOAP study [8] or the 7.7% estimated by Laakso et al. [12], both of which surveyed all scientific publishing. Therefore, it could be argued that HIV research is more open than research in other fields. Unfortunately, we do not have data to compare the HIV vaccine field to other areas of biomedical research.

Our survey of institutional subscriptions around the world resulted in widely varying levels of access, with researchers at some institutions having access to only 20% of subscription-based publications while researchers in other institutions had access to 90% of these publications. The WHO-sponsored HINARI access program at institutions in low-income countries, particularly in Sub-Saharan Africa, has undoubtedly had a very significant impact on publication access at these institutions, providing scientists in those countries access on par with that available at major research institutions in the North. It is important to note that HINARI is only available to not-for-profit and governmental institutions. However, it has been argued that for-profit companies in the private sector are also essential for the growth of scientific capacity in the developing world and for the development of products required for controlling diseases that preferentially affect the developing world [13]. Thus, an expansion of the HINARI model to local companies involved in biomedical research and development may be beneficial. Similar to the current country-level distinction between those who can afford subscriptions and those who can't, access through HINARI could be provided to small start-ups and biotech companies that are operating at a loss or with small profit margins.

Our results suggest that even well endowed research universities in the North lack access to all of the HIV vaccine research literature. Therefore, the research community must use other strategies besides an institutional subscription if they wish to access the entire body of literature. Anecdotal responses from researchers whom we contacted suggest that the research community uses a variety of approaches to access the scientific literature when they lack access at their own institutions (Table 1). Researchers sometimes contact colleagues whose institutions may have subscriptions to journals that their institution does not provide. Some use the login and passwords from other institutions - either officially, by virtue of an adjunct affiliation, or unofficially, by acquiring login information from colleagues. Some use the option to purchase a single article of interest, provided by many journals, while others opt for personal subscriptions to compensate for the lack of institutional subscription. For example, the Ivanovski Institute of Virology in Moscow does not subscribe to any English-language journals. When asked about procedures to access the English language literature, researchers at this and other institutes in Russia reported that it is common for heads of laboratories to purchase personal subscriptions to journals that they consider critical, while the rest of the literature is usually accessed through contacts with colleagues abroad, often facilitated by internet-based message boards. Thus, through a combination of approaches, researchers may be able to compensate for low levels of institutional subscription, but the time and energy necessary to obtain articles in this way undoubtedly puts an additional burden on those researchers who are already constrained in resources.

**Table 1:** Sample quotes from researchers, who were asked how they access papers, which require subscription, but are not available through subscription at their institution.

Ways to access	Quotes
Web forums	“When we need a paper, which we don't have access to, we ask our colleagues on web forum […] and they usually help”
Collaborations or joint affiliations	“we do use a login for a European university that one of our students was registered at”“I am fortunate that I still have my [US university] access” (from someone moving from US to an institute in Africa)“[We have] passwords for [Australian University] and [European University] because two of our doctors are from those institutions”“scientists in our labs … have access through their collaboration to the vast university library collections”
Colleagues at other institutes	“if I can't get a paper, I email friends overseas to download it for me”“I use my friends. I am lucky I have people who have sort of taken the habit in the last 6 months to forward me the things they download.”
Personal subscriptions	“I personally subscribe to three journals, which I then donate to the library of the institute”

Interestingly, contacting authors directly no longer appears to be a common approach to obtain reprints of publications. Our experience may provide some clues as to why this is the case. The response rate to reprint requests of only 50–60% and the not uncommon long delay in response make contacting authors a very unreliable and time-consuming way to access articles. Moreover, even though contact information of the corresponding author is usually available for recently published articles on the journal's website, it is frequently missing for older literature, creating a “catch 22” situation in which only those individuals with access to the paper online or in hard copy know the contact information of the corresponding author. Even when this information is available, it can be outdated. The decay of emails [14] contributed to the low-response rate we observed – out of 65 emails we checked, 8 could not be located and another 8 were outdated and bounced back. One potential solution to the problem of email decay is adoption of a system of unique IDs for researchers that allow tracking authors as they move from one institution to another or even completely leave the field.

The “green road to open access” relies on researchers archiving their final peer-reviewed drafts on personal websites or in institutional repositories [15]. Our study showed that only a very small number of researchers (2.5%) used this option, even though many journals allow this practice. Increasing the number of self-archived articles will depend on stronger institutional mandates requiring researchers to make their findings available, and on informing scientists of this opportunity as some may be unaware that it is allowed under contracts with many journals. The RoMEO database is a useful source of information that allows researchers to check quickly journal's policies regarding self-archiving [16]. The open-source EPrints software (www.eprints.org) allows anyone to setup a “green access” repository of scientific papers to which authors can contribute preprints and postprints. The software also simplifies the process of obtaining reprints from authors by providing an automated request to which the author can reply with a single click of a mouse. If widely adopted, this approach would save time and effort for both researchers in need of the manuscript, and authors, who would be more likely to respond to such requests.

The focus of this study was on HIV vaccine research, an area of biomedical research that is especially relevant to researchers working on global health issues and to researchers who live and work in those areas of the world preferentially affected by the AIDS epidemic. It would be of interest to investigate whether other areas of research that preferentially affect the developing world follow a similar pattern. While this study is small and preliminary, it reveals the complexity of the issue. We observed large differences in the level of subscription-provided access amongst institutions globally, but it is not immediately clear how these differences in access affect the conduct and pace of research at these institutions. Access to the latest results is essential for progress in science. Clearly, if scientists around the world are to contribute fully to the global efforts to control and eradicate diseases that preferentially affect the developing world, they must do so on as level a playing field as possible.
